# The Two Weapons against Bacterial Biofilms: Detection and Treatment

**DOI:** 10.3390/antibiotics10121482

**Published:** 2021-12-03

**Authors:** Adriana Cruz, Manuel Condinho, Beatriz Carvalho, Cecília M. Arraiano, Vânia Pobre, Sandra N. Pinto

**Affiliations:** 1 iBB—Institute for Bioengineering and Biosciences, Instituto Superior Técnico, University of Lisbon, Av. Rovisco Pais, 1049-001 Lisboa, Portugal; adriana.b.cruz@tecnico.ulisboa.pt; 2 i4HB—Institute for Health and Bioeconomy, Instituto Superior Técnico, University of Lisbon, Av. Rovisco Pais, 1049-001 Lisboa, Portugal; 3 Instituto de Tecnologia Química e Biológica António Xavier, Universidade Nova de Lisboa, Av. da República, 2780-157 Oeiras, Portugal; manuel.condinho@itqb.unl.pt (M.C.); beatriztdcarvalho@gmail.com (B.C.); cecilia@itqb.unl.pt (C.M.A.)

**Keywords:** bacterial biofilms, biofilm detection, biofilm imaging, antimicrobial treatment, antibiofilm agents

## Abstract

Bacterial biofilms are defined as complex aggregates of bacteria that grow attached to surfaces or are associated with interfaces. Bacteria within biofilms are embedded in a self-produced extracellular matrix made of polysaccharides, nucleic acids, and proteins. It is recognized that bacterial biofilms are responsible for the majority of microbial infections that occur in the human body, and that biofilm-related infections are extremely difficult to treat. This is related with the fact that microbial cells in biofilms exhibit increased resistance levels to antibiotics in comparison with planktonic (free-floating) cells. In the last years, the introduction into the market of novel compounds that can overcome the resistance to antimicrobial agents associated with biofilm infection has slowed down. If this situation is not altered, millions of lives are at risk, and this will also strongly affect the world economy. As such, research into the identification and eradication of biofilms is important for the future of human health. In this sense, this article provides an overview of techniques developed to detect and imaging biofilms as well as recent strategies that can be applied to treat biofilms during the several biofilm formation steps.

## 1. Introduction

Antimicrobial resistance is the inevitable consequence of prescribing antibiotics, and bacteria will continue to develop resistance to therapies. This is a critical problem in hospital environments in particular due to the low numbers of novel compounds or strategies under development [1]. Several factors contribute to this scarcity, including a market failure, produced by the lack of incentive for pharmaceutical companies to develop antibiotics. This lack of incentives for development of novel antibiotics is a result of a low return on investment, since they are fast-acting drugs, use of novel antibiotics is often reserved, and their use is ultimately unpredictable as resistance evolves [1].

A 2014 report commissioned by the U.K. government also predicted that millions of people are expected to die prematurely because of drug resistance over the next 35 years worldwide and the world’s gross domestic product (GDP) will be 2 to 3.5% lower than it otherwise would be in 2050 [2].

This problem is even more relevant when is associated with bacterial biofilms, since bacteria growing in biofilms show significantly reduced antibiotic susceptibility. For instance, implant-related infections are very hard to treat [3]. This is due to the fact that clinical procedures to treat implant-related infections involve replacement of the implant that can increase the risk for the patient developing severe complications, as well as the very high costs associated with this procedure [4]. 

Bacterial biofilms consist of densely packed communities that grow attached to surfaces and are responsible for the majority of human clinical infections [5,6]. Their resistance to conventional antibiotics is 10- to 1000-fold higher than that of planktonic bacteria [7].

This increased resistance can be attributed to different factors, including decreased diffusion of antimicrobial agents through the self-produced extracellular matrix (made of polysaccharides, nucleic acids, and proteins matrix) [8,9,10], altered metabolic activity, and formation of persister cells [11]. Moreover, a relevant characteristic of biofilms is the fact that bacteria are able to inter-communicate and collaborate to survive in even the hardest conditions. This cell–cell communication mechanism is known as the quorum sensing (QS) system [12]. Bacteria QS system consists of self-produced extracellular chemical signals (called autoinducers), and it can control important virulence factors such as biofilm formation and maturation, antibiotic resistance, bacterial swarming, and bacteria–host interaction [12,13].

Altogether, bacteria biofilms are hard to eradicate and can cause severe infections, and the increasing numbers of infections by antibiotic-resistant strains are expected to strongly impact the future of world economies.

In the light of this dark scenery, this review focuses on recent antibacterial and antibiofilm strategies and the state of the art of imaging techniques that are being used to study this bacterial lifestyle with the hope that the current situation can be altered in a near and brighter future. 

## 2. Imaging of Biofilms and the Diversity of Detection Methods

Currently, nuclear medicine imaging is still the standard technique for the detection of infectious diseases [14]. However, this technique has several drawbacks, including the fact that (i) it involves the exposition of the patient to radiation, (ii) it requires specialized equipment, and (iii) it requires operator training. 

As recognized, there is an urgency to develop more accurate diagnostic tools and treatment, particularly when bacterial infection progresses to biofilm. The noninvasive technique in clinical use does not offer an optimized approach to detect biofilm infection. The low resolution, low practicality, and impossibility to distinguish between bacterial infection and sterile inflammation are the reasons to develop new diagnostics tools for biofilm detection in clinical environments [15]. In the last few years, development of diagnostic approaches became urgent in order to improve the treatment of bacterial infections and preserve some medical procedures that need alternative tools to prevent and treat bacterial infections. 

Considering this, in this topic, we highlight some of the possibilities for the detection and imaging of bacterial biofilms.

### 2.1. Nuclear Imaging

Nuclear imaging has been applied in oncology and in infectious disease diagnostics. Some radionuclides such as technetium-99m, iodide-125, and indium-111 have been shown for years to be useful tools to radiolabeled compounds for medical applications [16]. 

However, some disadvantages are found in common bacterial imaging agents such as difficult radiochemical synthesis, non-specific adsorption, or small target receptor expression on bacteria of interest [16]. Despite these limitations, recent works have revealed that there are bacterial metabolites that can be radiolabeled and used as tracers to identify bacterial biofilm infections, such as the maltodextrin transport system [17,18]. 

The carbohydrate transport and metabolism has been reported as an essential tool for proliferation of bacteria in the human organism. Thus, there are some strategies that include carbohydrate metabolism as a target that can be very helpful in improving nuclear imaging in the field of infectious disease diagnostics. For instance, ^18^FDG (fluorodeoxyglucose) is one example of an important radiopharmaceutical that has been used for many years on positron emission tomography (PET). However, ^18^FDG as a contrast agent shows a high uptake in mammalian cells and absence of distinguishing bacterial infections from cancer or inflammation [19,20].

Although efforts are being made to find new radiopharmaceutical and contrast agents, there are some complications associated with radiochemical synthesis and low affinity/specify at the bacteria target. With the aim of increasing the sensitivity of currently imaging methods, researchers have developed other contrast agents targeting the bacterial carbohydrate metabolism. This is the case of ^18^F-maltohexaose (MH^18^F) [17]. MH^18^F nuclear imaging agent is internalized by a bacteria-specific maltodextrin transporter. Thus, the contrast agents conjugated with maltohexose were only internalized by bacterial cells and not by mammalian cells, which do not express maltodextrin transporters [21]. Moreover, one of the major advantages of maltodextrin-based compounds is their lower toxicity because they are widely used as food additives. The development of nuclear agents such as MH^18^F might be crucial to bacterial biofilm detection at an early stage. 

### 2.2. Ultrasound Contrast Agent Imaging

Ultrasonic imaging techniques and their combination with other methods have been explored in order to enhance the strategies to detect and quantify early and mature biofilms [22,23]. The acoustic approach has the advantage of monitoring the surface biofouling in real time, and it has been proved that the ultrasonic technique can monitor formation and growth of some microbiological colonies [22]. 

Ultrasound medical imaging has been developed with the addition of contrast agents, especially encapsulated gas bubbles, which has led to an improvement in medical diagnosis [24]. Another improvement was a novel design of ultrasound contrast agents (UCAs), including a target ligand to establish the difference between infectious and healthy tissue [25]. 

It is important to have a detection technique that allows for identification of biofilms in early stages because the late diagnostic of mature biofilms sometimes compromises their clinical treatment inside the human body. Echocardiography, for instance, has several limitations in the detection of intra-cardiac biofilm. *Staphylococcus aureus* is the most common isolate in infective endocarditis, and strategies have been developed that can evaluate and characterize mechanical and structural properties of its biofilm. In this sense, researchers have developed strategies that could be evaluated and that characterize mechanical and structural properties of *S. aureus* biofilm [26]; this is because *S. aureus* is the most common isolate in infective endocarditis [27]. *S. aureus* biofilm has been studied through a combination of targeted ultrasound contrast agents (UCAs) and fluorescent probes. In the first step, developed UCAs that bind a carbohydrate epitope allowed for a spatial scanning of biofilm structure by high-frequency scanning acoustic microscopy (SAM). A complementary analysis occurred with TRITC-labelled streptavidin by fluorescence microscopy. The merge between high-frequency acoustic scanning and fluorescence imaging allowed for the acquisition of spatial resolution and detection of the biofilm components [26]. The results obtained showed an improvement in biofilm diagnostics.

### 2.3. Optical Imaging and Probes

**Figure 1:**
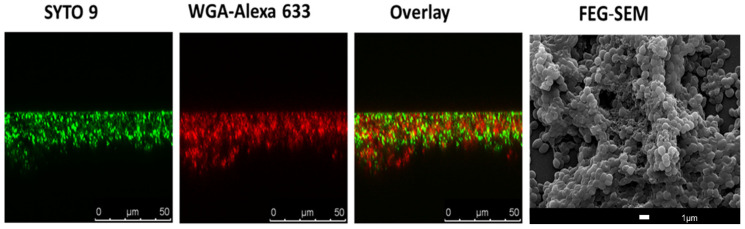
Imaging of bacterial biofilms with confocal scanning laser microscopy (CSLM) and SEM. Twenty-four hour *S. aureus* JE2 (MRSA) biofilm was co-labelled with SYTO 9 (green channel), a nucleic acid binding dye, and with WGA-ALEXA 633 (red channel), a wheat germ agglutinin dye that labels *S. aureus* biofilm matrix. The overlay between the two channels is also represented. In the right image, there is a representation scanning electron image (SEM) of 24 h *S. aureus* JE2 biofilms.

Optical imaging offers an important tool to understand/visualize the 3D biofilm structure. Multiple techniques are included in this range, such as scanning electron microscopy (SEM), confocal scanning laser microscopy (CSLM), light microscopy, infrared spectroscopy, and reflectance spectroscopy [28]. Figure 1 illustrates a *S. aureus* biofilm visualized with the use of CSLM and SEM.

SEM, a technique with high resolution that is based on surface scattering and absorption of electrons, has been used in different studies to visualize biofilms (Figure 1) since it is able to detect key structural components such as the presence of biofilm matrix [29,30]. Moreover, researchers have been using SEM to evaluate the efficacy of anti-biofilm compounds [31,32,33]. However, SEM is a very expensive technique, and quantitation of the biofilm is rather difficult, including the fact that researchers cannot work with live samples. 

Due to this, the most common used methodology to study the 3D biofilm morphology of biofilms is probably CSLM, and in fact, it is recognized that CSLM represents an important advance in technology-associated biofilm imaging [34]. In CSLM, due to the presence of a confocal pinhole, the out-of-focus fluorescent signals are eliminated [35], which is relevant when it is considered for instance with traditional fluorescent microscopy. Moreover, it allows for the formation of high-resolution images at different depths [36], which is crucial in biofilm studies. The tridimensional morphology and physiology of biofilms can then be screened by CLSM using a combination of molecular probes and fluorescent proteins optimized to target/visualize biofilm components. Most probes and fluorescent proteins are designed to stain cellular organelles and structures. However, in the last decades, there has been an effort in the development of proteins and fluorochromes to target, for instance, the extracellular matrix of biofilms. This includes the application of fluorescently labelled lectins (Figure 1) to visualize and characterize the biofilm matrix, in particular the extracellular polysaccharide components [37,38,39]. 

Extracellular DNA (eDNA) is also often a target for extracellular matrix imaging using CSLM. Propidium iodide, TOTO-1, and TO-PRO-3 iodide are probes that were used in this context, providing excellent distinction between biofilm eDNA component and the intracellular DNA found in biofilm cells [8,40]. These probes are often used in combination with SYTO 9 [8] or SYTO 60. SYTO 9 is a green fluorescent nucleic acid-binding dye. The fact that SYTO 9 is a membrane-permeable probe and TO-PRO-3 iodide or propidium iodide can only label cells with damaged membranes allows the viewer to discriminate between viable and nonviable cells [8].

Other fluorescent probes for extracellular DNA (eDNA) detection have recently been developed. This is the case of CDr15 probe, which was evaluated on *Pseudomonas aeruginosa* ΔwspF with a highly elevated cyclic-di-GMP content (mimicking the biofilm mode of growth) and a pYhjH strain with a low intracellular cyclic-di- GMP content (representative of the planktonic mode of growth). The results showed that CDr15 probes bind effectively to eDNA. The robustness of CDr15 as a diagnostic in vivo probe was evaluated on corneal infection model, and the results showed that biofilm regions were visualized after CDr15 treatment [41]. 

Identification of novel fluorescent probes together with targeting different biofilm structures will greatly facilitate diagnosis of biofilm infection. In this sense, a fluorescent probe, CDy11, that targets amyloid-like fibers in the *P. aeruginosa* biofilm matrix was developed. It was demonstrated that CDy11 allows for detection using in vivo imaging of *P. aeruginosa* in implant and corneal infection mice models [42]. In addition, CDy14 was identified as a potential fluorescent probe to target Psl exopolysaccharide in *P. aeruginosa* [43]. In this context, amphiphilic fluorescent carbon dots were developed and applied to assist the characterization of bacterial biofilm matrix [44]. The amphiphilic carbon dots (C-dots) were shown to readily bind to the EPS scaffold of *P. aeruginosa*, and it was detected for the first time as a dendritic morphology of the EPS.

Furthermore, the peptide nucleic acid fluorescence in situ hybridization (PNA FISH) technique has also been used to study biofilm’s structure and composition. Traditional FISH is a molecular technique on which labeled DNA probes hybridize to their complementary nucleic acid targets. The use of FISH (namely, DNA probes) to study microorganisms and biofilms can lead to some drawbacks, including poor target site specificity and poor signal-to-noise ratio [45,46]. The limitations associated with FISH can be overcome with the use of peptide nucleic acid (PNA) probes; PNA is a synthetic DNA analogue with a stronger binding to nucleic acids [47]. PNA FISH technique is very helpful for the CLSM observation of mixed biofilms since it allows for the use of multiple fluorescent probe labels that are characteristic of a specific microorganism [48,49]. 

Researchers have been using other methods besides PNA FISH in order to study complex biofilm structures such as those observed in mixed-biofilm populations. One method that is very popular involves the use of mutants expressing green fluorescent protein (GFP) and its variants [50,51,52,53,54,55]. The spatial-temporal fluorescence profiles of two or more fluorescent proteins constitutively expressed in biofilms was already successfully shown in biofilms prepared under dynamic flow conditions [56]. The combination of real-time CLSM imaging of biofilms expressing fluorescent proteins with flow systems allows for the acquisition of important and precise information regarding bacterial cell division and biofilm growth conditions and dynamics. Moreover, when biofilms are prepared under flow systems (including the use of microfluidic devices [57,58]), it is possible to obtain biofilms with a higher maturity degree such as mushroom-like structures found in *P. aeruginosa* biofilms [59]. Furthermore, the bond between the bacterial cells to the substratum surface is reported to be stronger when compared with static conditions [60]. The use of GFP and other variants in these real-time imaging studies is extremely helpful, but there are some artifacts caused by environmental factors that can have a negative impact in these assays, such as poor fluorescence at low pH and O_2_-dependence [56,61].

In vivo biofilm detection possesses a challenge for the scientific community. One promising approach for this purpose relies on the use of laser capture microdissection (LCM). Laser capture microdissection is a high-resolution technique that allows researchers to rapidly sample/isolate individual cells or cell compartments from solid tissue with the aid of a laser beam [62,63]. LCM has also been used to isolate non-cellular structures including amyloid plaques [64]. This microdissection technique is often used in cancer research, e.g., [65], and now researchers are using it to obtain information related with in vivo biofilms. For instance, very recently, the adaptation of *B. cereus* in *G. mellonella* gut infection model was demonstrated for the first time with LCM [66].

Another valuable imaging technique for identification of in vivo biofilms is target fluorescent imaging (TFLI). The principle of TFLI is targeting fluorophores that emit light outside the absorbance window of tissue in the near infrared region. There are some reports of targeting fluorescent imaging for tumor diagnostics, and the first clinical TFLI approach employment was observed in ovarian cancer surgery [67]. Furthermore, some studies have been published to also demonstrate the ability of TFLI for in vivo detection of bacteria [68,69]. Since TFLI emerged as useful tool for multiple diagnosis in clinical research, Marleen van Osteen and colleagues decided to combine the TFLI advantages with vancomycin’s well-known biodistribution profile. In this sense, the authors developed vanc-800CW as a new conjugate for optical biofilm imaging. For this propose the authors conjugated vancomycin with IRDye-800CW, a near-infrared fluorophore. The images were obtained by IVIS Lumina II imaging system [70]. 

The vanc-800CW potential as a fluorescent probe was evaluated in multiple models. The in vitro studies performed demonstrate a good detection for *Streptococcus* and *Dermabacter* species and minor detection of *Corynebacterium*. As expected, the results also confirmed the lack of vancomycin staining for Gram-negative bacteria such as *P. aeruginosa* and *Escherichia coli* [70]. 

To understand the potential of vanc-800CW in vivo model, the authors selected a mouse model of myositis induced by bioluminescent *S. aureus*. The administration of vanc-800CW allows for distinguishing between *S. aureus*-induced infection from *E. coli* induced-infection and sterile inflammation. The biodistribution profile also shows similarities with what is described for “native” vancomycin. A complementary post-mortem with contaminated implants was also performed to ensure the feasibility of BAI detection. The results were promising and confirmed the ability of vanc-88CW to stain *Staphylococcus epidermis*-containing implants. The fluorescent conjugated developed by Marleen van Osteen and colleagues displayed important and crucial results in biofilm imaging [70]. 

The application of carbon nanotube probes is another promising tool for in vivo targeting and fluorescence optical imaging of bacterial infections [71]. Using genetically engineered M13 virus as a multifunctional vector, Bardhan et al. synthesized NIR-II fluorescent SWNT probes, with additional functionalization on the virus for active targeting of bacterial infections [71]. The authors were able to successfully preform the detection of deep-tissue infective endocarditis using the SWNT probe. 

Both works [70,71] contributed positively to the challenge in the field of biomaterial-associated infection diagnostics and for non-invasive detection and monitoring of infectious diseases in the body.

### 2.4. Biofilm Detection with iTRAQ (Isobaric Tags for Relative and Absolute Quantitation)-Based Quantitative Proteomics Methods

As explained before, the biofilm structure contains several proteins that are important for its stability and maintenance [6]. The proteins present in the biofilm naturally depend not only on the type of pathogen but also on the developmental stage of the biofilm [72]. Therefore, identifying biofilm proteins can be a very useful biofilm detection method. For this purpose, isobaric tags for relative and absolute quantitation (iTRAQ)-based quantitative proteomics technique has been reported in several studies [73]. The iTRAQ technique allows for the identification and quantification of hundreds of proteins in different biological samples in one single experiment. It consists of the relative quantification with mass spectrometry of proteins in complex mixtures. iTRAQ technology uses isobaric reagents to label the primary amines of peptides and proteins [73]. During the iTRAQ process, reagents are reactive with amine groups, marking the sample peptides and maintaining the isobaric balance (sample mass does not change) [73,74]. An analysis of the reporter groups that are generated upon fragmentation in the mass spectrometer is then carried out. This procedure is commonly used to distinguish between normal and “diseased” samples and was also used to identify bacterial biofilm proteins [75,76]. Recently, an iTRAQ-based quantitative proteomics approach was used to identify protein markers associated with the biofilm formation of *Enterococcus faecalis* [77]. In this case, it was observed by iTRAQ that strong biofilm-forming clinical isolates have proteins associated with shikimate kinase pathway and sulfate transport upregulated. This is a relevant information since it can lead to the development of therapies that can act on these metabolic pathways, and consequently inhibit the biofilm formation of *Enterococcus faecalis* [77]. The iTRAQ technique has also been used to identify proteins present in biofilms that promote caries and other dental problems [78,79]. iTRAQ reporters determined that biofilm cells of *Tannerella forsythia* have upregulated oxidative stress response proteins, which is related with the fact that this sub-gingival pathogen is more resistant to oxidative stress, thus allowing it to persist in the oral cavity [79]. Thus, the iTRAQ-based quantitative proteomics technique can be very useful for biofilm detection and to find possible targets that could lead to biofilm eradication, as it allows for the understanding of which proteins and metabolic pathways are important for biofilm formation. 

### 2.5. The Use of Artificial Intelligence (AI) Technology for Biofilm Detection

Machine learning, together with image processing, has been employed in recent years to assist doctors during clinical and diagnostic process [80,81]. 

For biofilm detection, the use of machine learning models was already reported, e.g., detection of *E. coli* biofilm using an electro-chemical impedance spectroscopy (EIS)-based biosensor [82]. Machine learning systems, for instance, can be trained to recognize multiple impedimetric parameters and determine bacteria concentration. The conjugation of machine learning systems with EIS already showed promising results, even with thicker biofilm [82].

Convolutional neural network (CNN) has already been reported as a successful deep learning model for improving diagnostic field [83]. The CNN model is trained to learn visual patterns from images and has been used for medical images recognition [84,85]. Recently, this model was tested to improve a rhinocytology diagnostic exam [81,86]. For instance, it allowed for the detection of the presence of biofilm on rhino-cytological scans. The sample was stained, and cyan-colored spots were observed and were directly related with biofilm infection [86]. The cyan spots can vary with stage/maturity of the biofilm, and the CNN model system can be trained to recognize these patterns. The CNN model was also applied for detection of biofilm formation (all four stages) attached onto a metallic material. To achieve this purpose, the researchers trained the system to recognize the main features of the process on the basis of microscopy features. For *E. coli* strain, this mathematical model showed results in accordance with experimental detection of metal biofilm [87].

Moreover, the CNN deep learning model can also be trained to detect polymicrobial biofilm. Antoine Buetti-Dinh et al. reported a CNN model trained to detect a biofilm composed by *A. caldus* strain, *L. ferriphilum* strain, and *S. thermosulfi-dooxidans* of sulfide minerals. When compared to human experts, the CNN model showed a 90% of accuracy in contrast with 50%, thus offering an accurate alternative to classical and time-consuming biochemical methods [83].

## 3. Antibacterial and Antibiofilm Strategies

The difficulty of treating bacterial biofilm-associated infections, as explained above, is highly associated with the recalcitrant character of bacterial biofilm and the development of resistance toward antibiotics. Therefore, the development of strategies that allow for efficient inhibition of biofilm formation and/or to completely eradicate biofilms is one of the most challenging research topics of the present day. Understanding the mechanism behind biofilm formation is crucial to developing potential control strategies. This includes exploring potential targets against c-di-GMP, extracellular polysaccharide and eDNA present in biofilm matrix, and bacterial cell membrane and biofilm quorum sensing. In addition, ribonucleases and small non-coding RNAs (sRNAs) can also in the future be considered as potential targets since several research papers were able to demonstrate their importance in biofilm formation and regulation (e.g., [88]). In fact, ribonucleases were found to affect biofilm formation in several bacteria, such as *E. coli*, *Salmonella* Typhimurium, *P. aeruginosa*, and *Bacillus subtilis* [88,89,90,91]. These RNA-degrading enzymes affect biofilms by controlling the expression of biofilm matrix genes but also by modulating the levels of c-di-GMP and other biofilm regulators [92,93,94]. Other RNA regulators, namely, sRNAs, have been found to have a very important role in biofilm formation and antibiotic resistance [95,96]. However, thus far, no therapeutic drugs target these biofilm regulators, mainly due to the lack of basic knowledge on how exactly ribonucleases and sRNAs could be used to disrupt biofilms. 

In the context of therapeutic drugs, several compounds are currently being screened, including the antibody MEDI4893 and the antimicrobial peptide POL7080, both in clinical trial phase 2 [97]. Furthermore, in silico analysis or machine learning methods are becoming attractive strategies to help identify potential antibacterial and anti-biofilm inhibitory molecules [98,99,100]. In recent years, computational methods have emerged following the need for less consuming and more accurate results for the identification of antimicrobial and anti-biofilm molecules. In this context, some computational databases such as biofilm-active AMPs (BaAMPs) and tools/platforms including aBiofilm and Molib were created [101,102,103,104]. The aBiofilm platform has already provided the prediction of antimicrobial chemical molecules and their inhibitory activity [103]. Meanwhile, Molib tool, which is a training dataset of biofilm inhibitory molecules, has been shown to be even more accurate than the aBiofilm tool [104]. This focus on discovering small molecules with artificial intelligence might offer future solutions for the search of effective anti-biofilm drug discovery.

**Figure 2:**
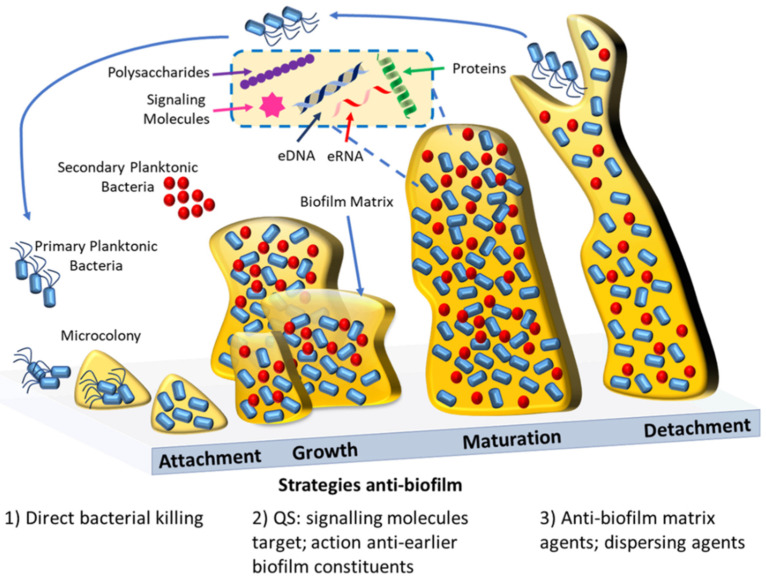
Schematic representation of biofilm formation and the current antibiofilm strategies. Biofilm formation starts with the initial reversible attachment of bacterial cells to a surface, then follows the growth of the biofilm within a matrix; maturation of the biofilm; and finally, when the environment conditions cease to be ideal, the reversal of the attachment with the dispersion of the cells that will colonize other superficies. Antibiofilm agents are capable of inhibiting the biofilm formation by bacteriostatic effects (1), e.g., antimicrobial polymers, or by acting against important early biofilm constituents (2), e.g., quorum sensing inhibitors. Mature biofilm can be disrupted by direct action against the biofilm matrix (3), e.g., biofilm matrix-degrading enzymes. Alternatively mature biofilms can be perturbed by the use of dispersing agents (3), e.g., nitric oxide.

In this section, we included examples of several other strategies and compounds that were developed in last years as potential antibacterial and anti-biofilm strategies (see also Figure 2). 

### 3.1. Linear and Cationic Polymers/Oligomers

Antimicrobial polymers have been shown to be promising alternatives to combat microorganisms [105,106]. In particular, cationic polymers have been widely explored and shown to be highly efficient in killing both Gram-positive and Gram-negative bacteria [105,107]. To achieve the best performance as antimicrobial agents, researchers have studied multiple polymers and developed them as mimic of antimicrobial peptides (AMP) but with a less expensive cost of production. 

The synthesis of polymers often considers molecular weight, cationic core, hydrophobicity, and architecture. The easily modification and structure optimization allows polymers to respond more accurately to bacterial target/metabolism characteristics. Molecular weight might influence diffusion of polymers across lipid membrane and contribute to bacterial cell death. To mimic AMP mechanism, the synthesis of polymers often includes a cationic group to interact with negative membrane of bacterial cells [106,108,109,110]. Quaternary ammonium groups or quaternary phosphonium group has been explored as a cationic core in new synthetic polymers [110]. Moreover, hydrophobicity is another important feature in new synthetized polymers to allow the polymers to cross the bacterial membrane. 

Madson and co-workers compared star-polymers and linear polymers [111]. The advantage of linear polymers is the feasibly and less complex synthesis. The authors synthetized polymers with (3- acrylamidopropyl)trimethylammonium chloride (AMPTMA), thus having a cationic and hydrophilic segment. To balance hydrophilic/hydrophobic profile, researchers chose n-butyl acrylate (n-BA) as the hydrophobic domain. In this work, the authors showed the potential of linear polymers as potent antimicrobial molecules and demonstrated them to be equally efficient as star-polymer [111]. This illustrated the fact that the polymer architecture itself is not always a requirement to improve antimicrobial activity. In all cases, cationic and hydrophobic groups of synthetic polymers were revealed to be determinant for the polymer’s antimicrobial activity. 

The antimicrobial potential of linear polymers or oligomers as AMP mimics was clearly demonstrated in several distinct works. For instance, against *E. coli*, linear polyethylemine (L-PEI) presents a minimum inhibitory concentration (MIC) value lower than branched PEI. For *E. coli* and *S. aureus*, L-PEI was shown to be able to permeabilize and disrupt bacterial cell membrane. In addition, the authors concluded that cationic and amphiphilic (hydrophobic and hydrophilic balance) are, as expected, crucial for antibacterial activity [112]. The potential of oxazoline-based antimicrobial oligomers was explored in two distinct works [105,106]. Using supercritical CO_2_, a method that reduces the use of toxic reagents, Correia et al. obtained biocompatible oxazoline-based oligomers quaternized with different amines [105]. The oxazoline-based oligomers showed a promising antimicrobial activity, and in particular, linear oligoethyleneimine hydrochloride (L-OEI-h) was able to kill several Gram-negative bacteria within a possible therapeutic range [105,106]. However, L-OEI-h was not very efficient in killing clinical isolates of *Klebsiella pneumoniae* producing KPC or OXA-48-like carbapenems [106]. This was attributed to the fact that the capsule of *K. pneumonia*, which is more “robust” than what was verified for other Gram-negative bacteria, may cause an efficient barrier against several antimicrobial agents, possibly including L-OEI-h. Thus, optimization of polymer design and synthesis can be crucial for eradication of infections caused by multidrug-resistant (MDR) bacteria, and this includes exploring different targets including biofilm quorum sensing. Linear polymers were already shown to target QS. For instance, Cavaleiro et al. described the synthesis of new linear polymers with methyl methacrylate as backbone and itaconic acid and methacrylic acid as monomers [113]. The authors found out that these compounds may interfere with bacteria quorum sensing. The results obtained in this study showed that these polymers decrease *Vibrio fisheri* bioluminescence, which is a process regulated by QS in this bacterium. The polymers were also efficient in inhibiting biofilm growth of *Aeromonas hydrophila* [113]. 

However, this approach directed against QS leads only to the dissociation of the biofilm architecture but not to the eradication of the biofilm. To ensure the total disruption of the biofilm is necessary to implement an additional treatment. This includes combination of several antimicrobial agents (e.g., antibiotics, membrane-lytic compounds such as AMP and cationic polymers) with anti-quorum sensing molecules. 

### 3.2. Nanoparticles 

Nanosized materials and nanoparticles in particular can play an important alternative to currently used antimicrobial agents or to be used as co-adjuvants. 

Nanoparticles including metallic nanoparticles, liposomes, and dendrimers appeared in response to the lack of antibiotics efficacy and drug resistance. These nanomaterials are very versatile, showing, for instance, the ability for encapsulation of antibiotics [114,115] and other antimicrobial agents. For example, it was recently demonstrated that the encapsulation of an antimicrobial glycolipid biosurfactant with the use of chitosan-based nanoparticles was effective against *Helicobacter pylori* biofilm [116]. When the use of nanoparticles (NPs) as effective antimicrobial agents is considered, some key properties are also taken into account, such as their large surface area relative to a small size volume and their excellent predisposition for surface modifications. The development of nanotechnology for antimicrobial propose is widely investigated at the pre-clinical level, and some nanoparticles are already approved by the FDA for clinical use (as in the case of liposomal formulations and lipid-based vaccines) or are in clinical trials [114].

As shown in several studies, nanoscale size allows for the nanoparticles to penetrate microbial cell walls and biofilms layers, causing several events such as irreversible damage of microbial membranes [9,117,118]. Several hypotheses for nanoparticles mechanisms of action have been explored, including cell membrane alterations and disruption [119,120], ROS generation, and lipid peroxidation [121,122,123]. Metabolic pathway disruption is also described as a mechanism of NPs [124,125]. 

As a good example of nanotherapeutics against biofilms, it is the application of polyoxometalates (POMs) supported by gold nanoparticles (AuNPs) on *P. aeruginosa* and *Staphylococcus* biofilms, which have been shown to have a potent effect in the inhibition of biofilms. This was attributed to the fact that AuNPs can induce a disruption of cell membrane integrity, leading to bacterial cell death [126]. 

In addition, NPs are also able to disturb the biofilm integrity by interacting with EPS, eDNA, proteins, and lipids of biofilms [118]. 

Polymer nanoparticles are the most successful nanoparticles used as antibacterial and antibiofilm strategies. For instance, nanostructures prepared from polymerization of monomers or from polymers were shown to represent a promising approach to overcome biofilm resistance through a controlled drug release strategy [115]. Relevant to this, it was concluded that in *P. aeruginosa* biofilm positively charged peripheral groups accumulate more efficiently into the biofilms [127], which is important information in terms of the design of drug-released nanoparticles, especially considering the presence of negatively charged molecules in the biofilm matrix. 

In a different approach, it was shown that cationic nano-engineered dendrimers were able to efficiently eliminate Gram-negative multi-drug-resistant bacteria both in vitro and in vivo systems, with very low toxicity towards human cells [128], and this was achieved without any resistance acquisition by MDR Gram-negative bacteria against their structurally nanoengineered dendrimers.

On the basis of the role of galactose-specific lectin LecA (PA-IL) and the fucose-specific lectin LecB (PA-IIL) on biofilm formation and the effect of deletion of lecA or lecB in biofilm formation, researchers put forward glycopeptide dendrimers as potential inhibitors of lectins LecA and LecB genes [129,130]. These dendrimers were evaluated by bioinformatic tools against *P. aeruginosa* biofilms. The results obtained highlight the importance of bioinformatics in this field. 

Metal-based nanoparticles also play an important role as an alternative against bacteria and antibiotic resistance. In fact, in the last few years, conventional metallic and biogenic nanostructures have received more attention as innovative antimicrobial agents, and, importantly, metal nanoparticles have an advantage in that they do not differentiate between resistant and susceptible bacteria [131]. The efficacy of simple and composite metal-based nanoparticles against planktonic and biofilm embedded bacteria have been discussed in several publications, including [132]. For instance, silver nanoparticles were shown to induce alterations in bacterial cell membranes, bacterial respiration, metabolism, and proliferation [133]. In addition, in *P. aeruginosa* biofilms, silver nanoparticles were able to inhibit quorum sensing signaling [134]. Oxide zinc nanoparticles (ZnO NPs) are also described to be potent weapons against biofilms [135], since they affect the stability of bacterial cell membrane, leading to bacterial death.

The antibiofilm potential of other metal-based nanoparticles as titanium dioxide NPs was also demonstrated [136]. Titanium-based nanomaterials have shown antibacterial activity against *Streptococcus mitis* and *S. aureus* [137], guided by initial electrostatic attraction and rapid killing of the microorganisms that indicates that the coating of medical surfaces with TiO_2_ NPs could prevent biofilm formation and reduce the contamination.

In addition, NPs also were described as antimicrobial coating agents with a potential clinical use in wound-dressing, catheters, bone cements, and cardiovascular implants [138].

### 3.3. Antibiofilm Photodynamic Therapy (PDT)

Photodynamic therapy (PDT) emerged recently in the field as an alternative therapy against biofilms [139,140,141]. PDT is based on employing a photosensitizer—a molecule/compound that produces cytotoxic ROS species after illumination with a specific wavelength light. The boost in ROS concentration has already shown a lethal effect in microbial pathogens. The main advantage of PDT against biofilms is the dual selectivity characteristic: a photosensitizer specially designed for infections and the adjustment of light wavelength to the lesion area [142]. 

In addition to the above, nanotechnology has been successfully used to improve the photosensitizer performance. This is the case of mesoporous silica nanoparticles (MSNs) (polymer-based nanoparticles), which were developed to be used as photosensitizer carrier. The malachite green was loaded into MSNs particles, and the efficacy of this nanoplatform was evaluated on Gram-positive and Gram-negative bacteria [143]. 

### 3.4. Nitric Oxide as an Agent against Biofilms

Nitric oxide (NO) is already reported as a signaling molecule involved in biofilm regulation, being also capable of participating in the regulation of cyclic di-GMP expression by the binding to H-NOX domains (heme-nitric oxide/oxygen-binding) [144,145]. At low concentrations, NO can make sessile microbes present in biofilm to initiate a motile life phase and consequently promote biofilm dispersion/disaggregation [145,146,147]. This approach involves NO donors that release NO and induce biofilm disruption. NO donors can be applied in different ways: as small molecules, incorporated into a polymeric delivery agent, or attached to a surface of polymeric or non-polymeric structure [148,149]. 

Despite the success of NO donors (e.g., SNP), there are some drawbacks already identified with this strategy, including the fact that the decomposition of some NO donors can release cyanide. In this sense, some alternatives to NO donors were explored, including the nitroxides. These are small molecules that possess a stabilized free radical and identical electronic properties to NO. Nitroxides can have an impact in biofilm growth but are not enough to kill bacteria directly. To overcome this issue, these compounds have already been used in combination with antibiotics for improvement of biofilm treatment and total eradication [150,151]. 

The potential of nitroxides on surface coating to prevent biofilm formation was also explored. For this, it was demonstrated that the use of polynitroxides, as a source of nitroxide, allow for the reduction of biofilm formation. In *P. aeruginosa* biofilm, the polynitroxide coating reduced the number of bacteria by up to 99%, an effect that was reported to be independent of concentration [152]. 

### 3.5. Biofilm Matrix-Degrading Enzymes

Biofilm matrix is composed of distinct elements including polysaccharides, proteins, and nucleic acids (eDNA and RNA) [153]. Several relevant functions are attributed to this biofilm structure, such as being a first line of defense against antimicrobial treatments [8]. Thus, many actual anti-biofilm strategies are designed to target the biofilm matrix and/or its components. This is the case of treatments that use biofilm-degrading enzymes. For instance, dispersin B, produced by the periodontal pathogen *Aggregatibacter actinomycetemcomitans*, was able to disintegrate mature staphylococcal biofilms. The enzyme acts by hydrolyzing the glycosidic linkages in the polysaccharide present in the staphylococcal biofilm matrix [154]. The effects of other enzymes are also reported for distinct biofilms. For instance, alginate lyase has been frequently used against the alginate polymer present in the *P. aeruginosa* biofilm matrix [155].

Some reports also showed that combined therapeutics results are an improvement in the anti-biofilm therapeutics. For instance, combination of DNase I (that denatures the eDNA) and dispersin B was found to inhibit staphylococcal skin colonization in an in vivo model [156]. Other studies were able to demonstrate the efficacy of combining biofilm-degrading enzymes with antimicrobial agents [157,158,159]. This is the case of meropenem and amikacin antibiotics that were combined with a trypsin/DNase I mixture against *S. aureus*–*P. aeruginosa* dual strain biofilms. The minimal biofilm eradication concentration (MBECs) values of both antibiotics were shown to decrease significantly when combined with the enzyme mixture [159].

Although being recognized as a potential relevant treatment in the fight against biofilms, the use of enzymes that degrade biofilm matrix is still very limited due to problems such as high cost associated with their production, handling procedures, and difficult large-scale production [159].

### 3.6. Targeting Amyloid-like Fibers 

For years, amyloid fibers were only associated with progressive human disorders including Alzheimer’s disease and type II diabetes. However, currently, they are recognized to play important biological roles in biofilm formation, thus being called in this case functional amyloid fibers [160]. When these amyloid-like fibers are correlated with the biofilm matrix, they can contribute to cell–cell and cell–surface interactions [161]. Moreover, in bacteria, the functional amyloid fibers can act as a protection barrier [162] or interfere with the function of specific proteins. In this sense, amyloid-like fibers represent interesting targets for the development of antibacterial and anti-biofilm drugs [163,164]. 

*B. subtilis* extracellular matrix contains two major components, an exopolysaccharide and the amyloid-like fibers formed by the TasA protein [165]. Thus, targeting TasA protein was demonstrated to be a possible alternative to conventional treatments [163]. The AA-861 and parthenolide compounds were shown to efficiently inhibit the formation of *B. subtilis* biofilms by inhibiting polymerization of the amyloid-like fibers of TasA [163], which reveals the importance of this amyloid-like fiber for the maturation of *B. subtilis* biofilms. Additionally, AA-861 and parthenolide were shown to have anti-biofilm activity against *E. coli*. The anti-biofilm mechanism was correlated with the inhibition of polymerization of Curli (the amyloid-like fiber present in *E. coli* biofilms) [163]. 

### 3.7. Targeting Functional Membrane Microdomains (FMM)

In the last decade, it was discovered that bacteria organize signal transduction, protein secretion, and transport processes in functional membrane microdomains (FMM), both in Gram-positive and Gram-negative cells [166]. This suggests that membrane organization and signaling transduction in bacteria is much more sophisticated than initially considered. Membrane domains in bacteria exhibit considerable heterogeneity, as evidenced by the detection of regions of increased fluidity (RIF). Curvature also regulates protein compartmentalization in bacterial membranes, as DivIVA and SpoVM have been, respectively, shown to display accumulation in sites of high negative (septation sites or poles) and positive membrane curvature (forespore membrane).

Considering the above, the FMMs are an attractive target to antibacterial agents [167]. 

Membrane domains with higher order were revealed in wild-type *B. subtilis*, and these were disrupted through the inhibition of polyisoprenoid lipid synthesis, suggesting that cyclic and noncyclic polyisoprenoids are likely to contribute to FMM formation. Inhibition of polyisoprenoid lipid synthesis also prevented biofilm synthesis in *B. subtilis* and *S. aureus*, revealing a possible link between these processes and FMM [167,168]. 

In *B. subtilis*, two different flotillin-like proteins, FloA and FloT, were found to insert in ordered FMM domains. Both of these proteins exhibit a punctate distribution dependent on polyisoprenoid lipids and cardiolipin (CL) [168]. FloA and FLoT were recently proposed to interact with a great number of different proteins, likely regulating their insertion in FMMs. Mutants lacking flotillins show severe dysfunction of diverse physiological processes, such as biofilm formation, natural competence, or sporulation [169,170].

### 3.8. Combination of Diagnosis and Treatment

The early detection of bacterial infections and tracing the issue of drug resistance with more efficient treatment are essential requisites to fight bacterial biofilms. In this sense, an interesting strategy for the selective treatment on the basis of drug resistance was recently proposed [171]. Paper-based devices (PBD) are promising platforms for antibacterial therapy. This methodology offers a sustainable, biosafety and low-cost alternative in the field of bacterial infections [172,173]. Furthermore, portable paper-based band-aids (PBA) were developed for sensing and treating drug resistance. A paper-based strategy was produced for drug-resistant bacteria, and it can test for the presence of β-lactamase-mediated resistance combined with selective antibacterial treatment after sensing drug resistance [171]. For detection of drug resistance, a colorimetric method can also be used [174]. In this case, drug-sensitive bacteria (DS) were detected by a color change from green to yellow. Then ampicillin-loaded nanomaterials (coated with chitosan) were able to eradicate DS bacteria. For drug-resistant (DR) bacteria, the colorimetric substrate nitrocefin can also be used. By the action of β-lactamase, which is secreted by many resistant bacteria, the subtract changed color from yellow to red. To treat drug-resistant bacteria, the authors developed porphyrin-based metal–organic frameworks (MOFs) that produce ROS after irradiation with light decreasing resistance to antibiotics [171]. The above elements were integrated together into cellulose paper, and the proof of concept was validated on DS and DR strains of *E. coli*.

## 4. Conclusions

Bacterial infection remains a challenge as result of the increasing number of antimicrobial-resistant strains. Consequently, bacterial infections are still responsible for high rates of mortality around the globe. This is in part due to the fact that a successful treatment is extremely hard to achieve once matured biofilms are established. They correspond to complex structures that can give bacteria strong hypothesis to survive, even under stress conditions (e.g., low amount of nutrients, low O_2_ levels, and acidic pH). Due to their structure, biofilms are difficult to study, and in this report, we have reviewed the state-of-the-art imaging techniques that are currently in use to study biofilms. We believe that an early detection of biofilms will be certainly helpful to treat patients and reduce healthcare costs. This can only happen through a continued improvement of the detection techniques and methodologies.

Furthermore, we explored several antibacterial and antibiofilm strategies (summarized in Table 1) that are being developed and that can only advance with the expansion of our scientific knowledge.

## Figures and Tables

**Table 1:** Antibacterial and antibiofilm strategies and their mechanism of action.

Antibacterial and Anti-Biofilm Strategies	Compounds	Mechanism of Action
Linear and cationic polymers/oligomers	Oxazoline-based antimicrobial oligomers (e.g., L-OEI-h)	Permeabilize and disrupt bacterial cell membrane.
	Linear polyethylemine (L-PEI)	Permeabilize and disrupt bacterial cell membrane.
	Linear polymers with methyl methacrylate as backbone, and itaconic acid and methacrylic acid as monomers	Interfere with QS systems, needs to be complemented with another antimicrobial agent.
Nanoparticles	Polyoxometalates (POMs) supported by gold nanoparticles (AuNPs)	Disrupt cell membrane integrity.
	Polymer nanoparticles	Efficient in disrupting biofilm matrix.
	Glycopeptide dendrimers	Potential inhibitors of lectins LecA and LecB genes.
	Silver nanoparticles	Induce alterations in bacterial cell membranes, bacterial respiration, metabolism, and proliferation. Moreover, inhibits QS signaling.
	Oxide zinc nanoparticles	Affect the stability of bacterial cell membrane.
	Titanium dioxide nanoparticles	Antibacterial and anti-biofilm proprieties guided by initial electrostatic attraction.
Photodynamic therapy (PDT)	Photosensitizer—a molecule/compound that produces cytotoxic ROS species after illumination with a specific wavelength light	ROS concentration increase leads to bacterial death.
Nitric oxide	Nitroxides	Affects biofilms but not sufficient to kill bacteria.
Biofilm matrix-degrading enzymes	Dispersin B	Hydrolyzes the glycosidic linkages in the polysaccharide present in the biofilm matrix.
	Alginate lyase	Lyses alginate from the biofilm matrix.
	DNase I	Denatures eDNA.
Targeting amyloid-like fibers	AA-861 and parthenolide	Inhibit polymerization of the amyloid-like fibers of TasA and Curli.
Targeting functional membrane microdomains (FMM)	FloA and FloT	Regulate the insertion of other proteins in FMMs.

## Data Availability

Not applicable.
